# Divergent genetic landscapes drive lower levels of AmpC induction and stable de-repression in *Serratia marcescens* compared to *Enterobacter cloacae*

**DOI:** 10.1128/aac.01193-23

**Published:** 2023-12-12

**Authors:** Jacob E. Lazarus, Yin Wang, Matthew K. Waldor, David C. Hooper

**Affiliations:** 1 Department of Medicine, Division of Infectious Diseases, Massachusetts General Hospital, Harvard Medical School, Boston, Massachusetts, USA; 2 Department of Medicine, Division of Infectious Diseases, Brigham and Women’s Hospital, Harvard Medical School, Boston, Massachusetts, USA; 3 Department of Microbiology, Harvard Medical School, Boston, Massachusetts, USA; 4 Howard Hughes Medical Institute, Boston, Massachusetts, USA; University of Fribourg, Fribourg, Switzerland

**Keywords:** AmpC, *Serratia marcescens*, *Enterobacter cloacae*, ceftriaxone, cefotaxime

## Abstract

The chromosomally encoded AmpC beta-lactamase is widely distributed throughout the Enterobacterales. When expressed at high levels through transient induction or stable de-repression, resistance to ceftriaxone, a commonly used antibiotic, can develop. Recent clinical guidance suggests, based on limited evidence, that resistance may be less likely to develop in *Serratia marcescens* compared to the better-studied *Enterobacter cloacae* and recommends that ceftriaxone may be used if the clinical isolate tests susceptible. We sought to generate additional data relevant to this recommendation. AmpC de-repression occurs predominantly because of mutation in the *ampD* peptidoglycan amidohydrolase. We find that, in contrast to *E. cloacae*, where deletion of *ampD* results in high-level ceftriaxone resistance (with ceftriaxone MIC = 96 µg/mL), in *S. marcescens* deletion of two amidohydrolases (*ampD* and *amiD2*) is necessary for AmpC de-repression, and the resulting ceftriaxone MIC is 1 µg/mL. Two mechanisms for this difference were identified. We find both a higher relative increase in *ampC* transcript level in *E. cloacae* Δ*ampD* compared to *S. marcescens ΔampDΔamiD2*, as well as higher *in vivo* efficiency of ceftriaxone hydrolysis by the *E. cloacae* AmpC enzyme compared to the *S. marcescens* AmpC enzyme. We also observed higher relative levels of transient AmpC induction in *E. cloacae* vs *S. marcescens* when exposed to ceftriaxone. In time-kill curves, this difference translates into the survival of *E. cloacae* but not *S. marcescens* at clinically relevant ceftriaxone concentrations. In summary, our findings can explain the decreased propensity for on-treatment ceftriaxone resistance development in *S. marcescens*, thereby supporting recently issued clinical guidance.

## INTRODUCTION

Beta-lactams, important and commonly used antibiotics, play a crucial role in the treatment of bacterial infections; they account for nearly two-thirds of worldwide antibiotic use (1). However, there are increasing rates of beta-lactam resistance, posing a threat to public health (2, 3). Among the multiple mechanisms of beta-lactam resistance in pathogenic Gram-negative bacteria, beta-lactamases are among the most prevalent and consequential (4). Most beta-lactamases are contained on mobile genetic elements, but chromosomally encoded beta-lactamases like the Ambler class C (AmpC) enzymes also play a vital role (5).

AmpC is widely distributed throughout the Enterobacterales, encoded by several potentially pathogenic species including the closely conserved enzymes in *E. cloacae*, *Klebsiella aerogenes*, and *Citrobacter freundii* (family Enterobacteriaceae), and the more divergent enzymes in *S. marcescens* and *Yersinia enterocolitica* (family Yersiniaceae) (6). Generally, AmpC enzymes exhibit high hydrolytic efficiency against penicillins and early generation cephalosporins like cefazolin and cephalexin, and lower efficiency against later generation cephalosporins like ceftriaxone, cefotaxime, and especially cefepime (6, 7).

Nevertheless, when AmpC is expressed at high levels, hydrolysis of ceftriaxone or cefotaxime can result in resistance (8). This high-level expression can be triggered by two mechanisms: transient induction or stable mutational de-repression. Transient induction occurs upon exposure to a beta-lactam antibiotic; the resulting derangement in cell wall remodeling causes a buildup in peptidoglycan fragments that bind to the AmpR transcriptional regulator, leading to increased *ampC* transcription (9 – 11). When cell wall homeostasis is restored, AmpC returns to basal levels. In contrast, stable de-repression of AmpC most often results from mutations in the peptidoglycan recycling pathway itself, especially in the peptidoglycan amidohydrolase AmpD. By this mechanism, AmpC remains expressed at high levels, and MIC values are often elevated, even in the absence of antibiotic (10, 12).

There is a concern that treating AmpC-producing Enterobacterales infections with ceftriaxone or cefotaxime, even if initial MIC testing indicates susceptibility, can lead to treatment failure due to either transient induction, mutational derepression, or both. On-treatment emergence of resistance has been observed in clinical series most frequently in *E. cloacae* and less so for *S. marcescens* (13 – 15). Recent analyses of *in vitro* mutation rates reveal considerably lower rates of the development of resistance to ceftriaxone in *S. marcescens* isolates compared to *E. cloacae* isolates (16). Based on these findings, there is now guidance that *S. marcescens* infections may be treated with ceftriaxone if the isolate tests susceptible, in contrast to infections with *E. cloacae* which should not (17).

However, the mechanisms responsible for the lower tendency for *S. marcescens* to develop ceftriaxone resistance remain poorly characterized. Possible explanations include variations in the amount of independent mutational events necessary for the emergence of ceftriaxone resistance, differences in the degree of AmpC overexpression required to produce resistance, or differential enzyme induction by ceftriaxone. Here, we investigate these potential mechanisms.

## RESULTS

We investigated differences in AmpC biology in the well-characterized type strains *E. cloacae* ATCC 13047 and *S. marcescens* ATCC 13880. We began by constructing comparable markerless deletion mutants and determining MICs by Etest for ceftriaxone and related beta-lactams as well as the unrelated agent tobramycin. Wild-type *E. cloacae* had a higher baseline ceftriaxone MIC than wild-type *S. marcescens* (Table 1). Expression of AmpC appears to be responsible for much of this difference since deletion of *ampC* in *E. cloacae* and in *S. marcescens* resulted in similar MICs. It has been previously shown using broth microdilution that in *S. marcescens*, like in the closely related *Yersinia enterocolitica* (18), deletion of both *ampD* and its orthologous peptidoglycan amidohydrolase *amiD2* is necessary for substantial elevation in ceftriaxone MIC. We confirm this result here using Etest gradient diffusion. Clinical & Laboratory Standards Institute (CLSI) breakpoints for “Intermediate” and “Resistant” correspond to a ceftriaxone MIC of 2 and 4 µg/mL, respectively. In wild-type *S. marcescens* ATCC 13880, deletion of *ampD* or *amiD2* alone results in small MIC elevations; deletion of both is necessary for an MIC of 1 µg/mL (representing a 16-fold increase from wild-type) (Table 1). It has been shown that OmpF is the *S. marcescens* porin most important for ceftriaxone permeation (19). In addition to *ampD* and *amiD2*, deletion of *ompF* is required for ceftriaxone resistance (with MIC = 8 µg/mL). In contrast, deletion of *ampD* alone in *E. cloacae* ATCC 13047 is sufficient for high-level ceftriaxone resistance (MIC = 96 µg/mL, representing a 128-fold increase from wild-type). Modest increases in cefepime and imipenem MICs were observed in *S. marcescens* and in cefepime MICs in *E. cloacae* mutants; however, all remained in the susceptible range. No substantial differences in tobramycin MICs were observed in either organism (Table 1).

**TABLE 1:** Minimum inhibitory concentrations (MIC) of wild-type and selected *S. marcescens* (SM) and *E. cloacae* (EC) deletion mutants^*a*^

MIC (μg/mL)	Ceftriaxone	Cefepime	Imipenem	Tobramycin
SM WT	0.064	0.023	0.38	1.5
SM Δ*ampC*	0.023	0.032	0.38	1.5
SM Δ*ampD*	0.25	0.064	0.38	1.5
SM Δ*amiD2*	0.125	0.032	0.38	1.5
SM Δ*ampD*Δ*amiD2*	1	0.094	0.38	1.5
SM Δ*ompC*	0.064	0.032	0.38	1
SM Δ*ompF*	0.25	0.25	0.5	1
SM Δ*ompC*Δ*ompF*	0.25	0.19	0.75	1
SM Δ*ampD*Δ*amiD*2Δ*ompC*	0.75	0.094	0.38	1
SM Δ*ampD*Δ*amiD2*Δ*ompF*	8	0.75	0.38	1
SM Δ*ampD*Δ*amiD2*Δ*ompC*Δ*ompF*	16	1	1	1
EC WT	0.75	0.047	0.25	0.75
EC Δ*ampC*	0.047	0.032	0.19	0.75
EC Δ*ampD*	96	0.5	0.25	0.75

^*a*^ MICs were determined by Etest. The values represent the mode of at least three individual biological samples.

We hypothesized that one contribution to these AmpC-dependent differences in ceftriaxone MIC could be the greater efficiency of the *E. cloacae* AmpC in ceftriaxone hydrolysis. The *k*_cat._/*K*_m_ (a measure of the catalytic efficiency for a given substrate) for the closely related cephalosporin, cefotaxime, is known to be about 10-fold higher in purified AmpC enzyme from *E. cloacae* compared to that from *S. marcescens* (7). To test this hypothesis *in vivo*, we performed scarless replacement of the *S. marcescens ampC* protein coding sequence with that of *E. cloacae*, and vice versa, preserving native upstream regulatory elements. We predicted that *S. marcescens* strains with the *E. cloacae ampC* would show increased MICs, and *E. cloacae* with the *S. marcescens ampC* would show decreased MICs. However, only a small increase in ceftriaxone MIC in *S. marcescens* with the *E. cloacae ampC* was observed (Table 2). Ceftriaxone MICs increased, as expected, in *S. marcescens* with *ampD* and *amiD2* deletion, but MICs were only slightly higher in those with the *E. cloacae ampC*. However, *E. cloacae* with *S. marcescens ampC* exhibited pronounced decreases in ceftriaxone MICs. This difference was particularly notable in *E. cloacae* Δ*ampD*; the strain with the *S. marcescens ampC* had a 16-fold lower ceftriaxone MIC than the strain with the native *E. cloacae ampC* (Table 2). These findings suggest that the higher hydrolytic efficiency of the *E. cloacae* AmpC for ceftriaxone contributed at least in part to the higher ceftriaxone MICs we observed.

**TABLE 2:** Minimum inhibitory concentrations (MIC) of wild-type and selected *S. marcescens* (SM) and *E. cloacae* (EC) knock-in and deletion mutants^*a*^

MIC (μg/mL)	Ceftriaxone	Cefepime	Imipenem	Tobramycin
SM WT	0.064	0.023	0.38	1.5
SM EC-*ampC*-KI	0.094	0.032	0.38	1.5
SM Δ*ampD*	0.25	0.064	0.38	1.5
SM EC-*ampC*-KI Δ*ampD*	0.38	0.032	0.38	1.5
SM Δ*ampD*Δ*amiD2*	1	0.094	0.38	1.5
SM EC-*ampC*-KI Δ*ampD*Δ*amiD2*	1.5	0.047	0.38	1.5
EC WT	0.75	0.047	0.25	0.75
EC SM-*ampC*-KI	0.19	0.032	0.25	0.5
EC Δ*ampD*	96	0.5	0.25	0.75
EC SM-*ampC*-KI Δ*ampD*	6	0.75	0.19	0.5

^*a*^ EC-*ampC*-KI denotes the *E. cloacae* AmpC protein coding sequence inserted into the *S. marcescens ampC* locus by allelic exchange, and vice-versa for SM-*ampC*-KI. MICs were determined by Etest. The values represent the mode of at least three individual biological samples.

Relative levels of de-repression of *ampC* expression could also contribute to AmpC-dependent differences in ceftriaxone MICs. To test this hypothesis, we performed reverse transcription relative quantitative real-time PCR (relative RT-qPCR) to compare wild-type *ampC* expression to those in de-repressed deletion mutants. As a negative control, we found, as expected, that *ampC* transcripts had very low abundance in the deletion mutants (Table 3). Similar to our MIC data which showed only small MIC increases in single *S. marcescens* amidohydrolase mutants, relative transcript levels were similar or only slightly increased compared to the wild-type in these strains. The *S. marcescens* double mutant had ~40 fold increased relative levels. In contrast, the *E. cloacae* single *ampD* deletion mutant had a marked ~500 fold increased relative level of *ampC* transcripts. Thus, *E. cloacae’s* greater relative *ampC* expression upon de-repression, as well as its higher intrinsic AmpC enzyme ceftriaxone hydrolytic efficiency likely both contribute to its increased resistance to ceftriaxone.

**TABLE 3:** Reverse transcription relative quantitative real-time PCR (RT-qPCR) of *ampC* in wild-type and selected *S. marcescens* (SM) and *E. cloacae* (EC) deletion mutants^*a*^

SM WT	Ref	EC WT	Ref
SM Δ*ampC*	0.06 ± 0.02	EC Δ*ampC*	<0.01
SM Δ*ampD*	0.91 ± 0.16	EC Δ*ampD*	510 ± 71
SM Δ*amiD2*	1.38 ± 0.35		
SM Δ*ampD*Δ*amiD2*	44.4 ± 8.6		

^*a*^ Values represent the mean of three individual biological samples, with standard errors, and are normalized to expression in the corresponding wild-type isolate.

Recently, Kohlmann et. al. determined species-specific mutation rates leading to cefotaxime resistance in clinical isolates with baseline susceptibility to cefotaxime (16). They observed 100-fold lower mutation rates in *S. marcescens* isolates compared to *E. cloacae* isolates. We predicted that one explanation for this finding is the greater number of mutations necessary for AmpC de-repression. Consistent with that hypothesis, we found that, despite a large inoculum of wild-type *S. marcescens*, we were unable to detect any ceftriaxone mutants after plating on 1 µg/mL ceftriaxone agar (Table 4). As expected, the mutant frequency was higher in wild-type *E. cloacae* for ceftriaxone concentrations of 1, 2, and 4 µg/mL. Deletion of both *ampD* and *amiD2* in *S. marcescens* was necessary for the recovery of a similar number of ceftriaxone mutants as in wild-type *E. cloacae*. Deletion of *S. marcescens ompC* and/or *ompF* alone had little influence on mutant frequency. Similar to their influence on MIC (Table 1), only amidohydrolase deletions in combination with porin deletions led to relatively high mutant frequencies. To ensure that mutant frequencies did not appear low in *S. marcescens* because of fitness defects in deletion strains, we compared their growth in rich and in minimal media; we did not detect differences in growth rates in either single or compound *S. marcescens* deletion mutants (data not shown).

**TABLE 4:** Mutant frequency of the indicated strain plated on the indicated ceftriaxone (CRO) concentration. At least three individual biological samples of *S. marcescens* (SM) and *E. cloacae* (EC) deletion mutants were grown as overnight cultures, diluted as detailed in Methods, and plated on ceftriaxone agar^*a*^

	CRO 1 ug/mL	CRO 2 ug/mL	CRO 4 ug/mL
SM WT	< 6E-10	ND	ND
SM Δ*ampD*	< 6E-10	ND	ND
SM Δ*amiD2*	2.8E-8 ±1.8E-8	< 6E-10	ND
SM Δ*ompC*	< 6E-10	< 6E-10	ND
SM Δ*ompF*	< 6E-10	< 6E-10	ND
SM Δ*ompC*Δ*ompF*	< 6E-10	< 6E-10	ND
SM Δ*ampD*Δ*amiD2*	1.3E-5 ±4.7E-6	7.6E-7 ±9.1E-8	1.1E-7 ±5.5E-7
SM Δ*ampD*Δ*amiD2*Δ*ompC*	ND	3.7E-7 ±2.0E-7	3.6E-8 ±7.4E-9
SM Δ*ampD*Δ*amiD2*Δ*ompF*	ND	1.7E-5 ±1.1E-5	4.7E-7 ±8.0E-8
SM Δ*ampD*Δ*amiD2*Δ*ompC*Δ*ompF*	ND	4.5E-5 ±6.6E-6	5.6E-7 ±1.6E-7
EC WT	6.6E-6 ±1.1E-6	1.8E-6 ±1.9E-7	8.6E-7 ±1.8E-7
EC Δ*ampD*	1.2 ± 0.1	0.9 ± 0.1	0.9 ± 0.1

^*a*^ The values represent the mean and standard error of the resulting colony counts, divided by the input CFU. ND, not determined. * <6E-10 indicates that no colonies were detected (out of a total inoculum of 1.7E9 CFU plated).

**Fig 1:**
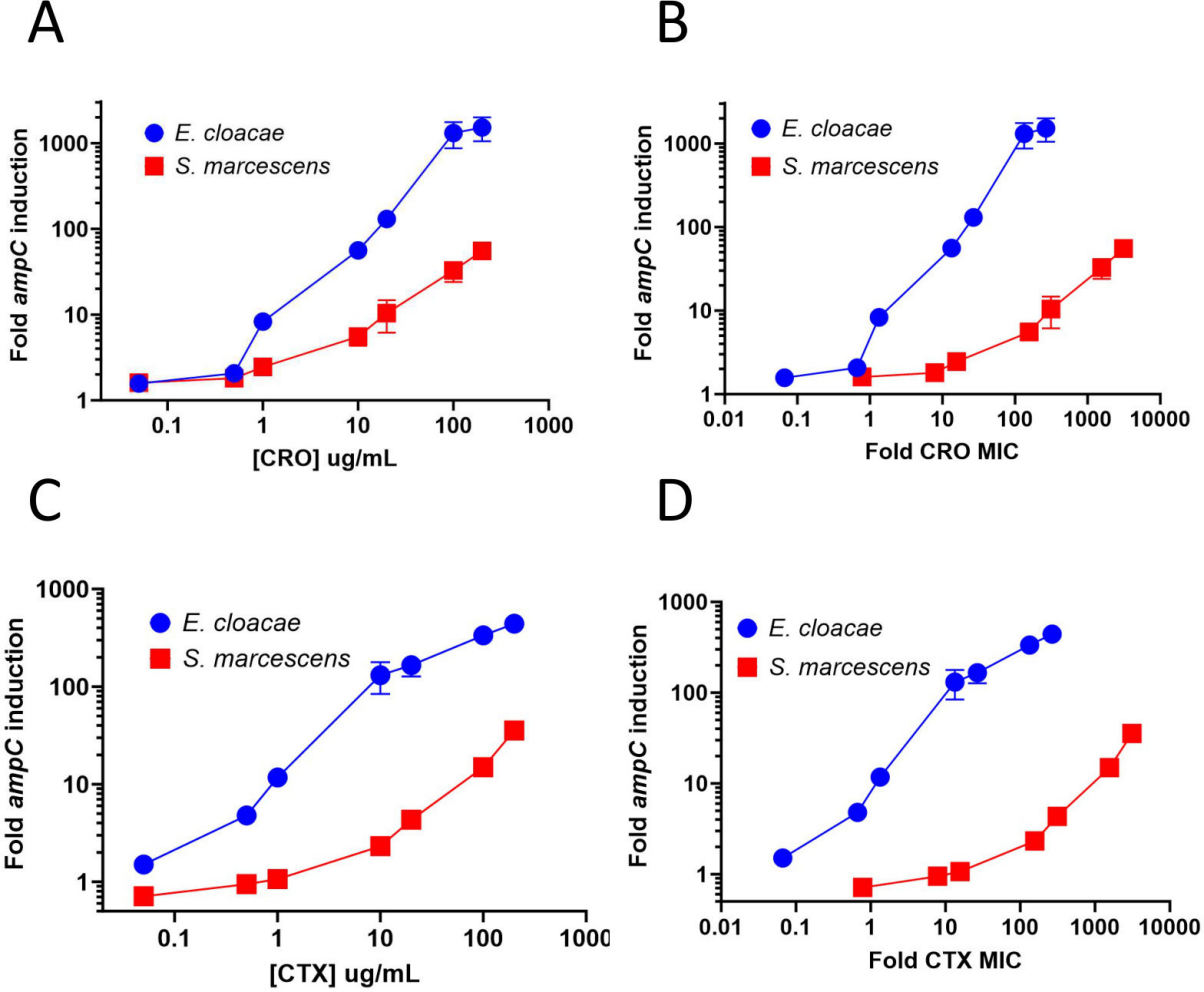
Reverse transcription relative quantitative real-time PCR (RT-qPCR) of *ampC* in wild-type *S. marcescens* and *E. cloacae* as a function of the inducing ceftriaxone (CRO) (**A**) or cefotaxime (CTX) (**C**) concentrations. Panels B and D are data replotted with the Y-axis expressed as a multiple of the MIC for the indicated antibiotic. Log-phase cultures were exposed to the indicated antibiotic concentration and samples isolated for processing after 1 hour. Values represent the mean and standard error of three independent biological samples.

The genetic selection and outgrowth of ceftriaxone mutants with stable de-repression of *ampC* may be facilitated *in vivo* by transient induction of *ampC* upon ceftriaxone exposure. In this way, transient increases in AmpC levels could support initial survival in ceftriaxone, supporting a sufficient inoculum for the appearance of de-repressed mutants. Though ceftriaxone and cefotaxime have historically been considered to be relatively poor inducers of *ampC* transcription (6), it has recently been shown that *E. cloacae* does undergo *ampC* induction at high concentrations of cefotaxime (that may be encountered *in vivo*) (20). We predicted that ceftriaxone might have similar potential for induction and sought to compare *ampC* induction in *S. marcescens* and *E. cloacae*. We exposed either wild-type *E. cloacae* or *S. marcescens* to therapeutic concentrations of ceftriaxone or cefotaxime (21 – 23) for 1 hour and determined the increase in relative levels of *ampC* transcription. We observed larger increases in relative *ampC* expression in *E. cloacae* compared to *S. marcescens* (Fig. 1A). At 10 µg/mL of ceftriaxone, *ampC* was induced at 10-fold higher levels in *E. cloacae* compared to in *S. marcescens,* and at 100 µg/mL of ceftriaxone, 40-fold higher levels were observed in *E. cloacae* compared to in *S. marcescens*. This difference was even more pronounced when *ampC* induction was normalized to baseline ceftriaxone MIC (Fig. 1B). Results were similar for cefotaxime (Fig. 1C and 1D ) and for ceftriaxone induction over 15 minutes as opposed to 1 hour (data not shown). Thus, ceftriaxone and cefotaxime stimulate greater induction of *ampC* expression in *E. cloacae* vs *S. marcescens*.

**Fig 2:**
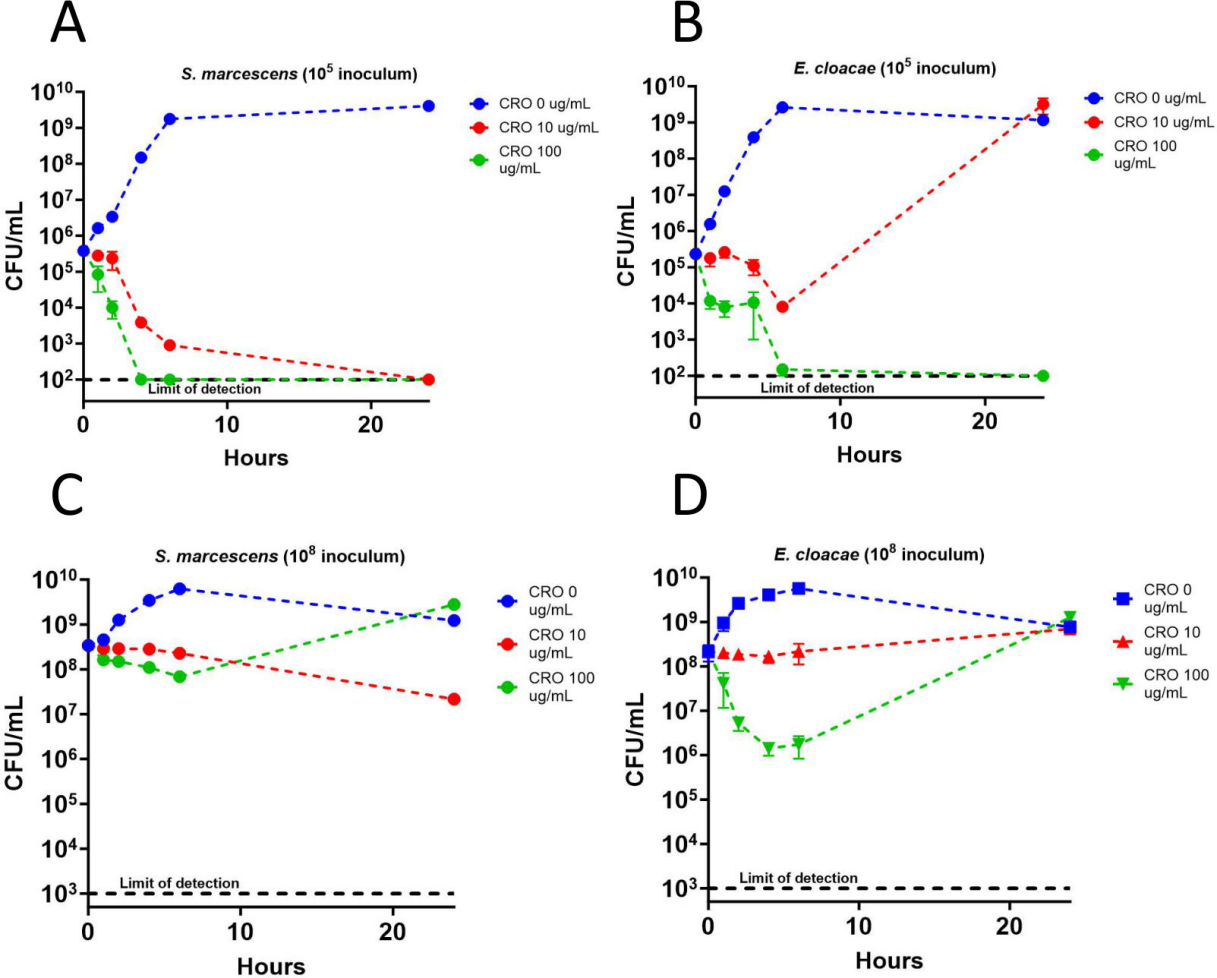
Time-kill assays. Log-phase *S. marcescens* or *E. cloacae* were adjusted to the indicated inoculum (either 10^5^ or 10^8^ CFU/mL) in either 0, 10, or 100 µg/mL ceftriaxone (CRO). Colony counts were performed on nonselective solid media for 24 hours. Values represent the mean and standard error of three independent biological samples.

We hypothesized that these large differences in ceftriaxone-triggered induction of *ampC* expression would allow wild-type *E. cloacae* to survive better than wild-type *S. marcescens* when exposed to ceftriaxone. We performed ceftriaxone time-kill curves with two different inocula to test this idea. A 10^5^ CFU/mL inoculum, typical of a urinary tract infection and higher than most blood stream infection (24, 25), and a 10^8^ CFU/mL inoculum that replicates the high local concentration of bacteria that might occur on a prosthetic device or in a heart valve vegetation (26) were tested. We used experimental ceftriaxone concentrations of 10 µg/mL and 100 µg/mL that are well above the MICs of both organisms, and would approximate achievable *in vivo* trough and peak concentrations, respectively (23). We found that at the 10^5^ CFU/mL inoculum, wild-type *S. marcescens* was killed at both ceftriaxone concentrations (Fig. 2A). In contrast, *E. cloacae*, after undergoing an initial decline in CFU at 6 hours after exposure to ceftriaxone 10 µg/mL, recovered to levels comparable to the no-ceftriaxone control by 24 hours (Fig. 2B). In the 10^8^ CFU/mL inoculum, both *E. cloacae* and *S. marcescens* had similar levels of viable bacteria compared to control by 24 hours at both ceftriaxone concentrations (Fig. 2C and 2D ).

## DISCUSSION

We have identified fundamental differences in the AmpC-related biology of two important Gram-negative pathogens, *E. cloacae* and *S. marcescens*. We find that *E. cloacae* has an increased propensity toward the development of ceftriaxone resistance due, at least in part, to two factors: the *E. cloacae* AmpC appears to have higher hydrolytic efficiency for ceftriaxone, and *ampC* transcript levels are an order of magnitude higher upon maximal de-repression. We also observed higher levels of ceftriaxone-stimulated AmpC induction in *E. cloacae;* these higher levels of enzyme, combined with its higher catalytic efficiency, led to increased survival of wild-type *E. cloacae* compared to *S. marcescens* in time-kill curve experiments.

Our findings generally fit well within the existing literature. Our measurement of lower ceftriaxone MICs in *E. cloacae* with its AmpC coding sequence replaced by that of *S. marcescens* is consistent with *in vitro* enzyme kinetics (7, 27, 28). Similar to our results with ceftriaxone, Guerin et al. observed comparable similar increases in cefotaxime MICs in an independently generated Δ*ampD* mutant, as well as comparable levels of *ampC* induction upon exposure to cefotaxime (20). Literature is sparse for *S. marcescens*; to our knowledge, ours are the first data quantitatively examining relative *ampC* transcript levels in response to experimental de-repression and to induction with ceftriaxone. The underlying mechanism responsible for lower levels of both de-repression and induction in *S. marcescens* remains unknown. Previous work has revealed that, in contrast to the Enterobacteriaceae, the *S. marcescens ampC* 5’ untranslated region has a stem-loop structure that serves to increase the half-life of the *S. marcescens ampC* transcript (29). This suggests that differences in transcript levels are presumably not due to transcript stability.

Our findings are in support of recent IDSA guidance that cautions against ceftriaxone use in general in *E. cloacae* infections (17). Given the lower levels of *ampC* induction, *ampC* de-repression, and the negligible rate of selection for ceftriaxone mutants, their recommendation that ceftriaxone may be used in *S. marcescens* infections when antimicrobial susceptibility testing indicates susceptibility is reasonable. However, our time-kill curves revealed poor killing of *S. marcescens* by ceftriaxone at high inocula. It thus may remain prudent, as suggested (17), to consider alternative treatment in *S. marcescens* infections with a lack of source control or the potential for biofilm formation (in which organisms are generally expected to respond relatively poorly to beta-lactams). This is not a rare situation; *S. marcescens* can be an exuberant biofilm former (30) and is an important cause of endocarditis, particularly in persons who use intravenous drugs (31 – 33), though in the largest case series of *S. marcescens* endocarditis to date, the microbiological cure rate with ceftriaxone (for susceptible isolates) was high (34).

Our studies have limitations. We chose *E. cloacae* ATCC 13047 and *S. marcescens* ATCC 13880 because they are the type strains for the species, but other strains may differ, particularly due to the substantial genetic heterogeneity observed in *S. marcescens* clinical strains (35). Relevant to this point, the lower baseline ceftriaxone MIC observed here in *S. marcescens* compared to *E. cloacae* could have important influences on the mutant frequencies observed. There are isolated reports where *S. marcescens* with higher baseline cefotaxime MICs have developed resistance through amidohydrolase mutation alone (in the absence of apparent porin deficits) (36, 37). However, baseline cefotaxime and ceftriaxone MICs generally tend to be low in *S. marcescens* clinical strains; in one collection of *S. marcescens* clinical isolates, only 6% tested ceftriaxone non-susceptible (38). In the past 5 years of SENTRY data, only 14% of submitted *S. marcescens* isolates tested non-susceptible compared to 23% of *E. cloacae* isolates (39). Additionally, our kill-curve experiments, while comprehensive, do not replicate *in vivo* conditions.

Future studies to uncover the mechanisms of differential *ampC* induction and de-repression between *S. marcescens* and *E. cloacae* will be worthwhile. It appears the additional AmpD ortholog in *S. marcescens* is unlikely to be an important factor, as we have found that deletion of both orthologs did not lead to similar transcript levels. If the *S. marcescens* AmpC has lower catalytic efficiency for ceftriaxone, as our experiments suggest, then one would predict an overall higher level of peptidoglycan turnover, leading to greater (not lower) levels of AmpC induction and derepression (12). Instead, we would speculate that the lower level induction of *ampC* expression in *S. marcescens* vs. *E. cloacae* may be due to differences in AmpR binding sequences, which are known to be divergent between these species (29), or perhaps due to lower affinity of peptidoglycan muropeptides for AmpR itself. Furthermore, it remains to be determined if instead of multiple amidohydrolase mutations, other single mutations can be selected *in vitro* and *in vivo* that result in resistance to ceftriaxone in *S. marcescens*. As an example, one recent clinical report implicated the Cpx envelope stress response in resistance (40).

In conclusion, we have described fundamental differences in the propensity for *E. cloacae* and *S. marcescens* toward AmpC de-repression and induction. These findings support clinical guidance, which should enable treatment of *S. marcescens* infections with narrower-spectrum beta-lactams, preserving cefepime and carbapenems for infections with organisms with higher risk of development of resistance to third-generation cephalosporins.

## MATERIALS AND METHODS

### Molecular biology

The accession numbers for the *S. marcescens* ATCC 13880 and *E. cloacae* ATCC 13047 genomic sequences used for primer design are CP072199.1 and CP001918.1, respectively. Allelic exchange using pTOX3 was used to make all in-frame deletions as described previously (41). 5’ and 3’ primers (which denote the deletion boundaries), the *E. cloacae* protein coding sequence 5’ and 3’ primers (used to amplify this to knock in to *S. marcescens*), and qRT-PCR primers are in Table 5. Primers were synthesized at Eton Bioscience (Boston, MA). The insert for the *E. cloacae ampC* (into the *S. marcescens* locus) was directly synthesized by Twist Bioscience (South San Francisco, CA)

**TABLE 5:** Primers used in this study. SM, *S. marcescens*. EC, *E. cloacae*

Deletion mutants	5' Primer	3' Primer
SM Δ*ampD*	CAGACACCTCTCTGCGGTGG	CGCTAATGACGCTGTTTACG
SM Δ*amiD2*	CGTAAAGTCCCTCTCTCGCT	ATGCCGAAACCGCCGCCGTT
SM Δ*ompF*	CTCATTGGTGTTATTCGGACAC	TAAGTTGTCTCGCTTAACGGC
SM Δ*ompC*	CGTTATTATCCTCGTTAATTATGTCGAGC	TAAGCATCAGCGTGTGAATG
SM Δ*ampC*	GATAGAGGCTCTTCGGTTGCAGG	CAAGCGTTGGATAAGCGCTGA
EC Δ*ampD*	GCTGGAACTCCTTATGTATGGTG	GCGTCGTCAGAATAAGGAGA
EC Δ*ampC*	CAGTTATCTTCCGTAATAGCGAG	CTACAGTAAACCTTTGCCGG
Knockin mutants		
EC *ampC*	ATGATGAAAAAATCCCTAAGCTGCGC	TTACTGTAGCGCGTCGAGGA
RT-qPCR		
SM *ampC*	CCGGTGAAATTGTCTCGTTT	CGGTAGAGCCGGTTTTGTTA
SM *mdh*	CCAGCTTCCTTCAGGTTCTG	GGCGTTAACGTTGAACAGGT
EC *ampC*	GGTACCGGATGATGTTACCG	AGCGTGTGGAACGTTTATCC
EC *mdh*	GCAGCCTACGGAAGTTGAAG	GCGTTCTGGATACGTTTGGT

### Mutant frequencies

To select the emergence of ceftriaxone-resistant mutants and calculate their frequency in different genetic backgrounds, we plated bacteria on a large area of solid agar containing ceftriaxone. In our preliminary experiments, we plated 500 µL of 10-fold diluted overnight culture on an individual Nunc Square BioAssay Dish (which has 500 cm^2^ of culture area) (ThermoFisher). Several strains with high baseline AmpC expression displayed a considerable inoculum effect when plated in this way, so higher dilutions were necessary when plating these strains. These dilutions were established in pilot experiments. Ultimately, for ceftriaxone 1 ug/mL agar dishes, overnight cultures were diluted as above for *S. marcescens* WT, Δ*ampD*, Δ*amiD2,* Δ*ompC,* Δ*ompF,* Δ*ompC*Δ*ompF,* and Δ*ampD*Δ*amiD2* 10-, 10-, 10-, 10-, 10-, and 40-fold respectively; *E. cloacae* WT and Δ*ampD* were diluted 100- and 200,000-fold respectively. For ceftriaxone 2 ug/mL agar dishes, *S. marcescens* Δ*amiD2,* Δ*ompC,* Δ*ompF,* Δ*ompC*Δ*ompF,* Δ*ampD*Δ*amiD2*, Δ*ampD*Δ*amiD2*Δ*ompC,* Δ*ampD*Δ*amiD2*Δ*ompF,* and *ΔampDΔamiD2ΔompCΔompF* were diluted 10-, 10-, 10-, 10-, 40-, 40-, 5,000-, and 5,000-fold. respectively; *E. cloacae* WT and Δ*ampD* were diluted 50- and 200,000-fold. respectively. For ceftriaxone 4 ug/mL agar dishes, *S. marcescens* Δ*ampD*Δ*amiD2*, Δ*ampD*Δ*amiD2*Δ*ompC,* Δ*ampD*Δ*amiD2*Δ*ompF,* and *ΔampDΔamiD2ΔompCΔompF* were diluted 20-, 20-, 80-, and 80-fold, respectively; *E. cloacae* WT and Δ*ampD* were diluted 20- and 200,000-fold, respectively. Colonies were quantitated after overnight incubation at 30°C to avoid plate overgrowth. Plating was performed for each experiment on four BioAssay Dishes, and the mean and standard error of the mean for mutant frequency were calculated from three independent overnight cultures.

### Minimal inhibitory concentration determination

MICs were determined using Etest per the manufacturer’s specifications (bioMerieux). The reported MIC is the mode of at least three separate determinations performed on separate days. If a mode could not be determined after the third sample, additional samples were tested to ensure accuracy.

### Time-kill kinetic assays

To determine the *in vitro* bactericidal activity of ceftriaxone and cefotaxime on wild-type *S. marcescens* and *E. cloacae*, overnight cultures of *S. marcescens* in LB media were diluted 30-fold and *E. cloacae* 25-fold in LB media. *S. marcescens* overnight cultures were diluted more to account for faster initial growth kinetics so as to ensure a similar initial inoculum. Then, cultures were incubated with shaking at 37°C. At 1 hour, either LB-alone or LB + ceftriaxone was added to yield final concentrations of 0, 10 ug/mL or 100 ug/mL ceftriaxone. Incubation with shaking was continued, and colony counts were performed at 1, 2, 4, 6, and 24 hours on non-selective LB agar to quantitate viable bacteria.

### Relative quantitative RT-PCR assays

To determine relative *ampC* mRNA transcript levels, total *E. cloacae* and *S. marcescens* RNA were isolated from log-phase cultures using the RNeasy Midi Kit (Qiagen). Procedures were generally according to the manufacturer’s directions with the following modifications to ensure satisfactory genomic DNA digestion: twice the volume of lysis buffer was used; DNA digestion was performed with twice the suggested DNAse concentration; and the incubation was extended to twice the suggested duration. cDNA was then synthesized from mRNA using the Verso cDNA synthesis kit (Abgene, ThermoFisher). Real-time reverse transcription relative quantitative PCR was then performed on the resulting cDNA using EvaGreen dye and the CFX96 Real-Time System (Bio-Rad). The housekeeping gene *mdh* was used to determine ΔCT values for both *E. cloacae* and *S. marcescens*. Three technical replicates were performed for each reaction and subsequently averaged. The graphed values result represent the mean and standard error of the mean from three biological replicates performed on three different days. PCR primers are listed in Table 5. Primer efficiency was established using serial 10-fold dilutions of template DNA. Relative RT-PCR was then performed for each primer pair. The slope of the best-fit line for the trend between log(dilution) and average cycle threshold was then calculated. Finally, primer efficiency was calculated using the formula: *Efficiency* (%) = (10^(-1/slope)^−1)*100. All primer pairs had efficiencies that averaged between 102% and 105%.
